# Polyoxomolybdate Layered Crystals Constructed from a Heterocyclic Surfactant: Syntheses, Pseudopolymorphism and Introduction of Metal Cations

**DOI:** 10.3390/ma15072429

**Published:** 2022-03-25

**Authors:** Jun Kobayashi, Keisuke Shimura, Keisuke Mikurube, Saki Otobe, Takashi Matsumoto, Eri Ishikawa, Haruo Naruke, Takeru Ito

**Affiliations:** 1 Department of Chemistry, School of Science, Tokai University, Kanagawa 259-1292, Japan; j.koba92@gmail.com (J.K.); dohc.supertracker@gmail.com (K.S.); k_m0423_tokai@yahoo.co.jp (K.M.); sob0406qwer@gmail.com (S.O.); 2 Application Laboratories, Rigaku Corporation, Tokyo 196-8666, Japan; t-matumo@rigaku.co.jp; 3 Department of Applied Chemistry, College of Engineering, Chubu University, Aichi 487-8501, Japan; eishikawa@isc.chubu.ac.jp; 4 Chemical Resources Laboratory, Tokyo Institute of Technology, Kanagawa 226-8503, Japan; hnaruke@gmail.com

**Keywords:** polyoxometalate, inorganic-organic hybrid, layered crystal, surfactant, metal cation

## Abstract

Crystals with layered structures are crucial for the construction of functional materials exhibiting intercalation, ionic conductivity, or emission properties. Polyoxometalate crystals hybridized with surfactant cations have distinct layered packings due to the surfactants which can form lamellar structures. Introducing metal cations into such polyoxometalate-surfactant hybrid crystals is significant for the addition of specific functions. Here, polyoxomolybdate–surfactant hybrid crystals were synthesized as single crystals, and unambiguously characterized by X-ray structure analyses. Octamolybdate ([Mo_8_O_26_]^4–^, Mo_8_) and heterocyclic surfactant of 1-dodecylpyridinium (C_12_py) were employed. The hybrid crystals were composed of α-type and β-type Mo_8_ isomers. Two crystalline phases containing α-type Mo_8_ were obtained as pseudopolymorphs depending on the crystallization conditions. Crystallization with the presence of rubidium and cesium cations caused the formation of metal cation-introduced hybrid crystals comprising β-Mo_8_ (C_12_py-Rb-Mo_8_ and C_12_py-Cs-Mo_8_). The yield of the C_12_py-Rb-Mo_8_ hybrid crystal was almost constant within crystallization temperatures of 279–303 K, while that of C_12_py-Cs-Mo_8_ decreased over 288 K. This means that the C_12_py-Mo_8_ hybrid crystal can capture Rb^+^ and Cs^+^ from the solution phase into the solids as the C_12_py-Rb-Mo_8_ and C_12_py-Cs-Mo_8_ hybrid crystals. The C_12_py-Mo_8_ hybrid crystals could be applied to ion-capturing materials for heavy metal cation removal.

## 1. Introduction

Layered materials comprise two-dimensionally piled chemical components showing distinct structural anisotropy [1]. Such two-dimensional anisotropy induces characteristic properties such as conductivity [2,3], intercalation [4], magnetism [5], or emission capability [6]. Crystalline layered materials possess merits regarding their thermal stability and structural ordering in the long range, which can improve their properties [7,8,9,10]. In addition, introduction of metal cations into the crystalline layered materials can provide another function such as uptake of heavy or toxic metal cations [11,12,13].

To construct functional layered materials, inorganic polyoxometalate (POM) anions [14,15,16,17,18] and surfactant cations [19,20] represent useful components. The molecular-designable POMs and lamellar-forming surfactants can build up precisely controlled layered materials in their structures and functions [21,22,23,24,25,26,27]. Selective introduction of metal cations into the POM–surfactant hybrids can enable the emergence of a desired function such as ionic conductivity or metal cation-capture.

**Scheme 1:**
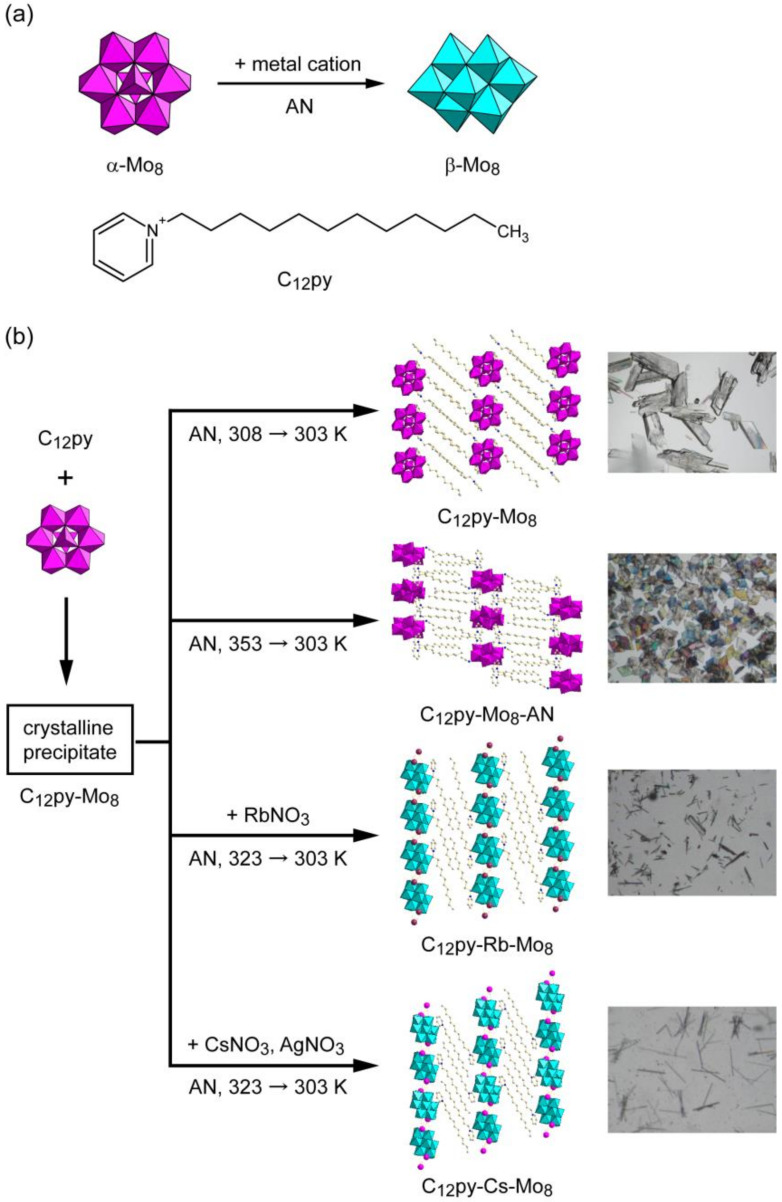
(**a**) Molecular structure of utilized components. Upper: α- and β-octamolybdate (α- and β-Mo_8_) anions isomerizing in acetonitrile (AN) under the presence of metal cation. Bottom: 1-dodecylpyidinium (C_12_py) cation; (**b**) Schematic procedures of the syntheses of C_12_py-Mo_8_ and related hybrid crystals. Some colorless crystals of C_12_py-Mo_8_-AN were colored in the photograph due to attached polarizing filters.

Among several POM–surfactant hybrid systems, POM–surfactant hybrid crystals can tune their crystalline ordered structure and function by selecting a combination of POM anion and surfactant cation [28,29,30,31,32,33,34,35,36,37,38]. Several metal cations (Na^+^, K^+^, Ag^+^, etc.) have been incorporated into polyoxomolybdate hybrid crystals [39,40,41], which were accompanied with the isomerization of octamolybdate ([Mo_8_O_26_]^4^^–^, Mo_8_) (Scheme 1a). The isomerization often occurs in an acetonitrile solvent [42]. These isomers in the solid state can be identified by infrared (IR) spectra [43,44]. Using heterocyclic pyridinium surfactant is effective for synthesis of Mo_8_-surfactant hybrid crystals incorporating metal cations. However, the examples have been limited to crystals consisting of 1-hexadecylpyridinium ([C_5_H_5_N(C_16_H_33_)]^+^, C_16_py) [39,40,41], which were obtained in low yields (< 10%) and sometimes in a mixed phase [41].

Here, several Mo_8_-surfactant hybrid crystals were successfully obtained by using 1-dodecylpyridinium surfactant ([C_5_H_5_N(C_12_H_25_)]^+^, C_12_py, Scheme 1a). The shorter alkyl chain of C_12_py is considered to enhance the solubility and crystallization of C_12_py-Mo_8_ hybrid crystals, and indeed enable the synthesis of several C_12_py-Mo_8_ hybrid crystals in a pure phase and a higher yield (>~30%). In this report, syntheses and pseudopolymorphism of the C_12_py-Mo_8_ hybrid crystals were investigated, and the introduction of metal cations into the C_12_py-Mo_8_ hybrid crystals was achieved with Rb^+^ and Cs^+^. The temperature dependence of the yield was evaluated for metal cation-introduced C_12_py-Mo_8_ hybrid crystals to assess the possibility of capturing heavy or radioactive metal cations.

## 2. Materials and Methods

### 2.1. Genaral Procedures and Instrumental Methods

All chemical reagents were purchased from FUJIFILM Wako Pure Chemical Corporation (Osaka, Japan) and Tokyo Chemical Industry Co., Ltd. (TCI, Tokyo, Japan) and utilized without further purification.

Infrared (IR) spectra were measured on a Jasco FT/IR-4200ST spectrometer (KBr pellet method). Powder X-ray diffraction (XRD) patterns were recorded with a Rigaku MiniFlex300 diffractometer (Cu Kα radiation, *λ* = 1.54056 Å) at ambient temperature. CHN (carbon, hydrogen and nitrogen) elemental analyses were carried out with a PerkinElmer 2400II elemental analyzer. X-ray fluorescence (XRF) analyses were performed with a Hitachi EA1000AIII XRF analyzer.

### 2.2. Syntheses of C_12_py-Mo_8_ and Related Hybrid Crystals

#### 2.2.1. C_12_py-Mo_8_

Na_2_MoO_4_∙2H_2_O (1.5 g, 6.2 mmol) or (NH_4_)_6_Mo_7_O_24_∙4H_2_O (1.0 g, 0.81 mmol) was dissolved in H_2_O (10 mL), and 6M HCl was added to adjust the pH to 3.8. To this solution was added a water/ethanol (10 mL, 1:1 (*v/v*)) solution of C_12_pyCl∙H_2_O (0.81 g, 2.7 mmol) and stirred for 10 min. The resulting suspension was filtered and dried in dark conditions to obtain colorless crystalline precipitate of C_12_py-Mo_8_ (0.90–1.2 g, yield 55–70%) (Scheme 1b). An acetonitrile (AN) solution (15 mL) containing the C_12_py-Mo_8_ precipitate (0.03 g) was heated at 308 K for one day, and sequentially the resulting colorless supernatant was stored at 303 K to obtain colorless plate crystals of C_12_py-Mo_8_ (yield ~30%) (Scheme 1b). CHN elemental analysis: Calcd for C_72_H_132_N_4_Mo_8_O_28_: C: 38.11, H: 5.86, N: 2.47%. Found: C: 38.68, H: 5.74, N: 2.52%. IR (KBr disk): 943 (s), 913 (s), 845 (m), 804 (m), 777 (m), 714 (s), 686 (m), 664 (m), 646 (m), 556 (w), 520 (m), 487 (w), 472 (w), 454 (w), 443 (w), 412 (w) cm^–^^1^. The single crystals of C_12_py-Mo_8_ were also obtained by gradual reoxidation of reduced molybdenum POM species (C_12_py-red-Mo, see Supplementary Materials).

#### 2.2.2. C_12_py-Mo_8_-AN

An acetonitrile (AN) solution (15 mL) containing the crystalline precipitate of C_12_py-Mo_8_ (0.03 g) was heated at 353 K for 3 h. The resultant colorless supernatant was kept at 303 K to obtain colorless plate crystals of C_12_py-Mo_8_-AN (yield ~30%) (Scheme 1b). Better single crystals were crystallized from the C_12_py-Mo_8_ precipitate synthesized with (NH_4_)_6_Mo_7_O_24_·4H_2_O. Solvent molecules of crystallization were easily removed under ambient atmosphere. CHN elemental analysis: Calcd for C_68_H_120_N_4_Mo_8_O_26_: C: 37.51, H: 5.56, N: 2.57%. Found: C: 37.21, H: 5.37, N: 2.70%. IR (KBr disk): 953 (w), 910 (s), 847 (m), 805 (s), 720 (w), 662 (s), 556 (w), 505 (w), 477 (w), 457 (w), 421 (w) cm^–^^1^.

#### 2.2.3. C_12_py-Rb-Mo_8_

An acetonitrile (AN) solution (15 mL) containing the crystalline precipitate of C_12_py-Mo_8_ (0.03 g) and solid RbNO_3_ (0.02 g) was heated at 323 K for 1 day. The resulting colorless supernatant was kept at 303 K to obtain colorless needle crystals of C_12_py-Rb-Mo_8_ (yield ~25%) (Scheme 1b). The yield of the C_12_py-Rb-Mo_8_ crystals was estimated based on the mass of added RbNO_3_ by changing the keeping temperature (279, 288, 293, 303 K). XRF analysis confirmed the atomic ratio of Rb:Mo to be 2:8. CHN elemental analysis: Calcd for C_34_H_60_N_2_Rb_2_Mo_8_O_26_: C: 22.06, H: 3.27, N: 1.51%. Found: C: 21.86, H: 3.04, N: 1.51%. IR (KBr disk): 951 (s), 942 (s), 903 (s), 844 (s), 725 (s), 680 (m), 653 (m), 578 (w), 555 (w), 524 (w), 479 (w), 448 (w), 409 (w) cm^–^^1^.

#### 2.2.4. C_12_py-Cs-Mo_8_

Colorless needles of C_12_py-Rb-Mo_8_ were obtained by a similar procedure for C_12_py-Rb-Mo_8_ (yield ~10%) (Scheme 1b). Solid CsNO_3_ (0.02 g) and AgNO_3_ (0.02 g) were employed instead of RbNO_3_. The yield of the C_12_py-Cs-Mo_8_ crystals was estimated based on the mass of added CsNO_3_ by changing the keeping temperature (279, 288, 293, 303 K). XRF analysis confirmed the atomic ratio of Cs:Mo to be 2:8. CHN elemental analysis: Calcd for C_34_H_60_N_2_Cs_2_Mo_8_O_26_: C: 20.98, H: 3.11, N: 1.44%. Found: C: 20.88, H: 3.11, N: 1.58%. IR (KBr disk): 953 (m), 942 (s), 906 (s), 848 (m), 773 (w), 732 (m), 659 (m), 553 (w), 525 (w), 475 (w), 434 (w) cm^–^^1^.

### 2.3. X-ray Crystallography

Single crystal X-ray diffraction data for C_12_py-Mo_8_ were measured on a Rigaku XtaLAB P200 diffractometer by using graphite monochromated Mo Kα radiation. The diffraction data were collected with CrystalClear [45] and processed with CrysAlisPro [46]. Diffraction data for C_12_py-Mo_8_-AN and C_12_py-Rb-Mo_8_ were collected and processed on a Rigaku R-AXIS RAPID diffractometer by using graphite monochromated Mo Kα radiation with PROCESS-AUTO [47]. Diffraction data for C_12_py-Cs-Mo_8_ were measured on a Rigaku Saturn70 diffractometer using multi-layer mirror monochromated Mo Kα radiation. The diffraction data were collected with CrystalClear and processed with CrysAlisPro.

Crystal structures except for C_12_py-Mo_8_-AN were solved by SHELXT (Version 2014/5 or 2018/2) [48], and the structure of C_12_py-Mo_8_-AN by SIR92 [49]. The refinement procedure was performed by the full-matrix least-squares using SHELXL (Version 2018/3) [50] through CrystalStructure software package [51]. Non-hydrogen atoms were refined anisotropically, and the hydrogen atoms on carbon atoms were located in calculated positions.

## 3. Results

### 3.1. Syntheses of a Series of C_12_py-Mo_8_ Hybrid Crystals

**Figure 1:**
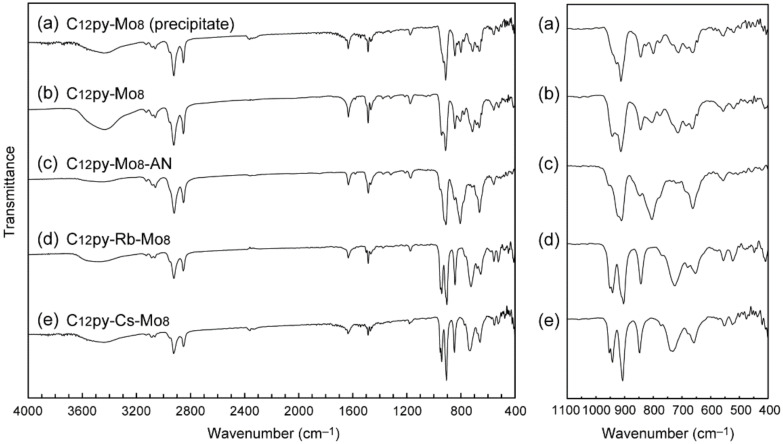
IR spectra of C_12_py-Mo_8_ and related hybrid crystals: (**a**) C_12_py-Mo_8_ precipitate; (**b**) C_12_py-Mo_8_ after recrystallization; (**c**) C_12_py-Mo_8_-AN; (**d**) C_12_py-Rb-Mo_8_; (**e**) C_12_py-Cs-Mo_8_.

**Figure 2:**
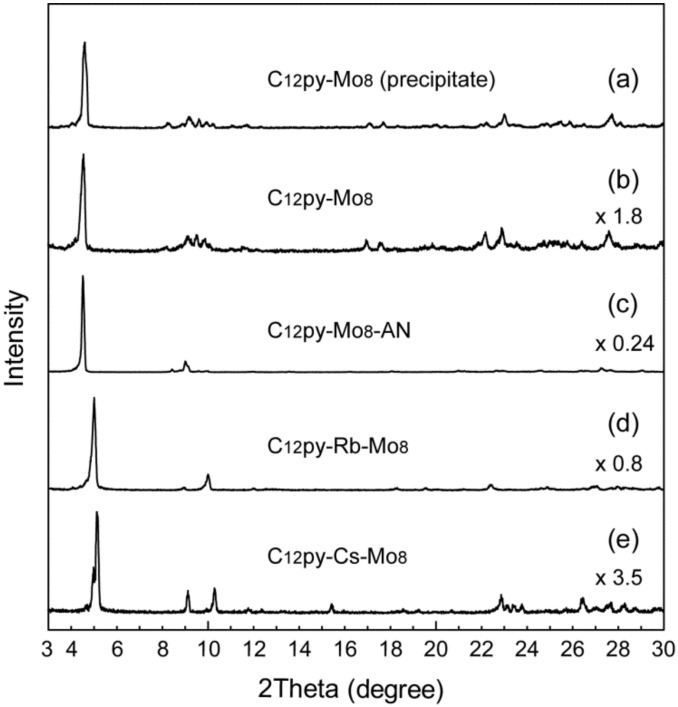
Powder XRD patterns of C_12_py-Mo_8_ and related hybrid crystals: (**a**) C_12_py-Mo_8_ precipitate; (**b**) C_12_py-Mo_8_ after recrystallization; (**c**) C_12_py-Mo_8_-AN; (**d**) C_12_py-Rb-Mo_8_; (**e**) C_12_py-Cs-Mo_8_.

Crystalline precipitate of C_12_py-Mo_8_ was obtained in 55–70% yield (based on Mo) by the reaction using acidified aqueous solution (pH = 3.8) of Mo_8_ species and C_12_py cation. The IR spectrum of C_12_py-Mo_8_ precipitate (Figure 1a) shows characteristic peaks owing to α-Mo_8_ [42,43,44] in the range of 400–1000 cm^–^^1^ together with the peaks of C_12_py (pyridine ring in 1400–1500 cm^–^^1^ and methylene groups in 2800–3000 cm^–^^1^), demonstrating the successful hybridization of α-Mo_8_ anion and C_12_py cation. The peaks in the 910–960 cm^–1^ range could be assigned to terminal Mo=O vibrations relevant to MoO_6_ unit [52,53,54]. The peaks in the 400–880 cm^‒1^ range could be due to Mo‒O‒Mo vibrations. The peaks around 805 cm^‒1^ may be attributed to Mo‒O vibrations of MoO_4_ unit [53]. The obtained C_12_py-Mo_8_ precipitate was highly crystalline as judged from the powder XRD pattern (Figure 2a) and did not depend on the difference in the molybdenum sources. The C_12_py-Mo_8_ precipitate was successfully recrystallized as single crystals from the acetonitrile solution (Scheme 1b), which was supported by similar IR spectra (Figure 1a,b) and powder XRD patterns (Figure 2a,b) of C_12_py-Mo_8_ before and after the recrystallization.

Another hybrid crystal of C_12_py-Mo_8_-AN was obtained from the starting C_12_py-Mo_8_ precipitate under different crystallization conditions (different heating temperature of 353 K, Scheme 1b). The IR spectrum of C_12_py-Mo_8_-AN (Figure 1c) indicates the presence of the α-Mo_8_ anion hybridized with the C_12_py cation. However, both IR spectrum and powder XRD pattern (Figure 2c) for C_12_py-Mo_8_-AN were slightly different from those of C_12_py-Mo_8_ (Figure 1a,b and Figure 2a,b), suggesting the formation of a different crystalline phase from C_12_py-Mo_8_. The emergence of a different phase was revealed by single crystal structure analyses (see below).

Hybrid crystals of C_12_py-Rb-Mo_8_ and C_12_py-Rb-Mo_8_ were obtained under the presence of Rb^+^ and Cs^+^ cations (Scheme 1b). Their IR spectra exhibit characteristic peaks of β-Mo_8_ (400–1000 cm^–^^1^) [42,43,44] as well as the peaks of C_12_py (1400–1500 and 2800–3000 cm^–^^1^) as shown in Figure 1d,e, indicating that the β-Mo_8_ anion existed in the obtained hybrid crystals. This implies that the isomerization of α-Mo_8_ to β-Mo_8_ occurred during the crystallization process [41], which was supported by single crystal structure analyses (see below). The peaks in the 910–960 cm^–1^ and 400–880 cm^–1^ range could be attributed to terminal Mo=O and Mo‒O‒Mo vibrations of MoO_6_ units in β-Mo_8_, respectively [52,53,54]. The distinct peaks around 655 and 730 cm^−1^ may suggest the presence of a two-dimensional connection between β-Mo_8_ anions and metal cations [42,43,44].

For all these hybrid crystals, the measured powder XRD patterns were similar to those calculated from the single crystal structure analyses (Figure S1). This demonstrates that each C_12_py-Mo_8_ hybrid crystal was obtained essentially as a pure phase. C_12_py-Cs-Mo_8_ may contain a minor phase with a different layer distance due to the different alkyl chain conformation in C_12_py. Slight differences in the reflection peak position and intensity of the measured and calculated patterns will be derived from the different measurement temperatures (powder: ambient temperature, single crystal: 93 or 193 K), and from preferred orientation due to the distinct layered structures.

### 3.2. Crystal Structures of C_12_py-Mo_8_ Hybrid Crystals

**Figure 3:**
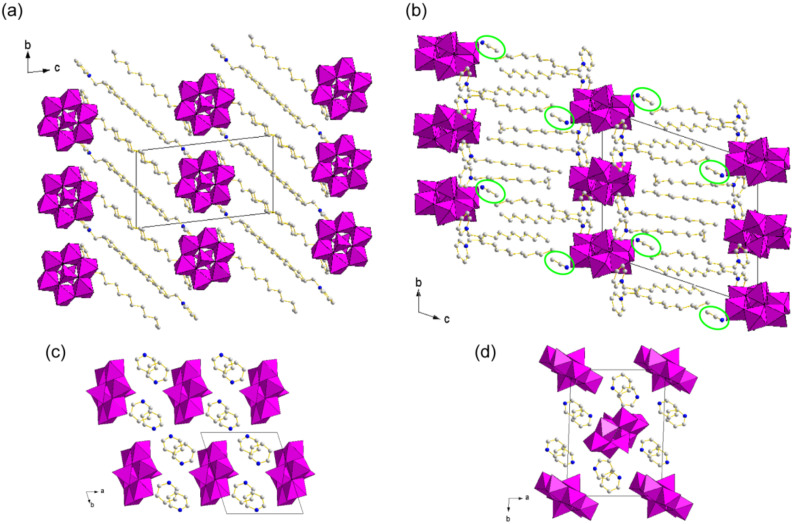
Crystal structures of C_12_py-Mo_8_ and C_12_py-Mo_8_-AN (C: gray, N: blue; α-Mo_8_ in purple polyhedrons). H atoms and disordered atoms are omitted for clarity: (**a**) Packing diagram of C_12_py-Mo_8_ along the *a* axis; (**b**) Packing diagram of C_12_py-Mo_8_-AN along the *a* axis. Some solvent molecules are highlighted by green ovals; (**c**) Molecular arrangements of C_12_py-Mo_8_ in the inorganic layers (*ab* plane); (**d**) Molecular arrangements of C_12_py-Mo_8_-AN in the inorganic layers (*ab* plane).

Crystalline precipitate of C_12_py-Mo_8_ was successfully recrystallized with hot acetonitrile as mentioned above. The formula was revealed to be [C_5_H_5_(C_12_H_25_)]_4_[α-Mo_8_O_26_] by the single crystal X-ray and CHN elemental analyses (Table 1). Four C_12_py cations (1+ charge) and one α-Mo_8_ anion (4‒ charge) were associated to compensate the opposite charges. Another type of counter cation or solvent of crystallization was not included. C_12_py-Mo_8_ exhibited a distinct layered structure consisting of α-Mo_8_ monolayers and C_12_py interdigitated bilayers with a periodicity of 19.0 Å (Figure 3a). This C_12_py-Mo_8_ phase was also obtained by gradual reoxidation of C_12_py-red-Mo (Table S1, Figures S2 and S3).

Crystalline phase of C_12_py-Mo_8_-AN was obtained from the same starting precipitate of C_12_py-Mo_8_ under the different heating temperature (Scheme 1b). The formula of C_12_py-Mo_8_-AN was [C_5_H_5_(C_12_H_25_)]_4_[α-Mo_8_O_26_]∙CH_3_CN, which consisted of one α-Mo_8_ anion, four C_12_py cations, and one additional acetonitrile of crystallization (Figure 3b). The compositional difference between C_12_py-Mo_8_-AN and C_12_py-Mo_8_ was only in the presence of crystallization solvent, which means that the hybrid crystal of C_12_py-Mo_8_-AN was a pseudopolymorph of C_12_py-Mo_8_. C_12_py-Mo_8_-AN contained alternate stacking of α-Mo_8_ monolayers and C_12_py bilayers with an interlayer distance of 19.4 Å. The acetonitrile molecules of crystallization were located at the interface between the α-Mo_8_ and C_12_py layers

Each α-Mo_8_ anion in C_12_py-Mo_8_ and C_12_py-Mo_8_-AN was isolated by the pyridine rings inserted into the inorganic layers (Figure 3c,d). There was one crystallographically-independent α-Mo_8_ anion in C_12_py-Mo_8_, while there were two crystallographically-independent α-Mo_8_ anions in C_12_py-Mo_8_-AN, resulting in the different molecular conformation of α-Mo_8_ in the C_12_py-Mo_8_ and C_12_py-Mo_8_-AN hybrid crystals (Figure 3c,d). This difference and the presence of acetonitrile in the vicinity of α-Mo_8_ may lead to a slightly different IR spectrum of C_12_py-Mo_8_-AN (Figure 1c) from C_12_py-Mo_8_ (Figure 1a,b) [55].

### 3.3. Crystal Structures of Metal Cation-Introduced Hybrid Crystals Derived from C_12_py-Mo_8_

**Figure 4:**
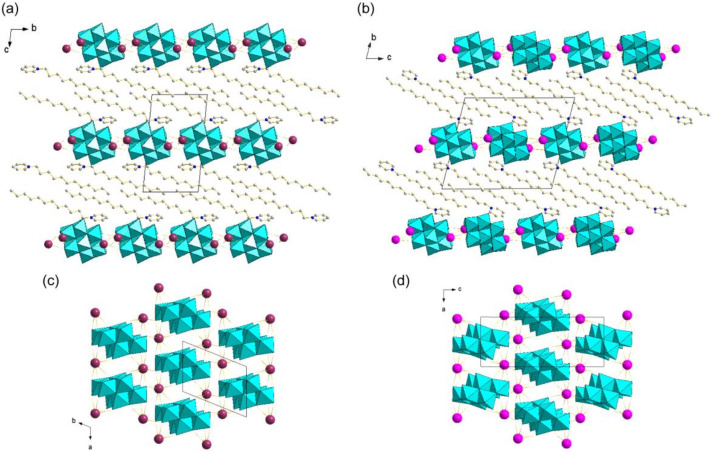
Crystal structures of C_12_py-Rb-Mo_8_ and C_12_py-Cs-Mo_8_ (C: gray, N: blue, Rb: plum, Cs: pink; β-Mo_8_ in blue polyhedrons). H atoms and disordered atoms are omitted for clarity: (**a**) Packing diagram of C_12_py-Rb-Mo_8_ along the *a* axis; (**b**) Packing diagram of C_12_py-Cs-Mo_8_ along the *a* axis; (**c**) Molecular arrangements of C_12_py-Rb-Mo_8_ in the inorganic layers (*ab* plane); (**d**) Molecular arrangements of C_12_py-Cs-Mo_8_ in the inorganic layers (*ac* plane).

Crystallization of the C_12_py-Mo_8_ precipitate under the presence of metal cations led to the formation of hybrid crystals in which the corresponding metal cation was introduced (Scheme 1b). Rb^+^ and Cs^+^ were successfully incorporated into the hybrid crystals as shown in Table 1 and Figure 4. The chemical formulae of C_12_py-Rb-Mo_8_ and C_12_py-Cs-Mo_8_ were [C_5_H_5_N(C_12_H_25_)]_2_Rb_2_[β-Mo_8_O_26_] and [C_5_H_5_N(C_12_H_25_)]_2_Cs_2_[β-Mo_8_O_26_], respectively. Both metal cation-introduced hybrid crystals contained the β-Mo_8_ anion as indicated by the IR spectra (Figure 1d,e). Two C_12_py cations (1+ charge) and two Rb^+^ and Cs^+^ were associated with one β-Mo_8_ anion (4− charge) due to charge compensation. No solvent molecule was included in the crystal structures.

The C_12_py-Rb-Mo_8_ and C_12_py-Cs-Mo_8_ hybrid crystals were composed of alternately stacked β-Mo_8_ monolayers and C_12_py interdigitated bilayers. The layer periodicities were 17.5 Å for C_12_py-Rb-Mo_8_ and 16.8 Å for C_12_py-Cs-Mo_8_, respectively (Figure 4a,b). The inorganic layers consisted of the β-Mo_8_ anions connected by Rb^+^ or Cs^+^, which held nine-fold coordination environment through terminal and bridging O atoms of three β-Mo_8_ anions (Figure 4c,d). The bond distance was 2.85–3.21 Å (mean value: 3.03 Å) for Rb–O, and 3.06–3.41 Å (mean value: 3.21 Å) for Cs–O, respectively. The pyridine rings of C_12_py were excluded from the inorganic layers formed by the densely connected β-Mo_8_ anions and metal cations [41].

Although the C_12_py-Rb-Mo_8_ and C_12_py-Cs-Mo_8_ hybrid crystals held similar structures, there were some subtle differences. C_12_py-Rb-Mo_8_ had one crystallographically-independent β-Mo_8_ anion, and C_12_py-Cs-Mo_8_ had two crystallographically-independent β-Mo_8_ anions, leading to the different molecular conformation of β-Mo_8_ in the inorganic layers (Figure 4c,d). As for the conformation of the C_12_py surfactant, the dodecyl chains were partly interdigitated in the C_12_py-Rb-Mo_8_ hybrid crystals, while fully interdigitated in C_12_py-Cs-Mo_8_ (Figure 4a,b). This may lead to the longer interlayer distance of C_12_py-Rb-Mo_8_ (17.5 Å) than C_12_py-Cs-Mo_8_ (16.8 Å).

### 3.4. Yields of Metal Cation-Introduced Hybrid Crystals Relecant to Ion-Capturing Property

Metal cation-introduced hybrid crystals of C_12_py-Rb-Mo_8_ and C_12_py-Cs-Mo_8_ were obtained from the starting C_12_py-Mo_8_ hybrid crystals (Scheme 1b). The coexisting C_12_py-Mo_8_ hybrid crystal and metal cation in the crystallization solution will induce the formation of C_12_py-Rb-Mo_8_ and C_12_py-Cs-Mo_8_. This means that the C_12_py-Mo_8_ hybrid crystal can capture Rb^+^ and Cs^+^ from the solution into the solid phase as the C_12_py-Rb-Mo_8_ and C_12_py-Cs-Mo_8_ hybrid crystals. Therefore, the yield of C_12_py-Rb-Mo_8_ and C_12_py-Cs-Mo_8_ can be related to the ion-capturing property of C_12_py-Mo_8_ hybrid crystals.

**Figure 5:**
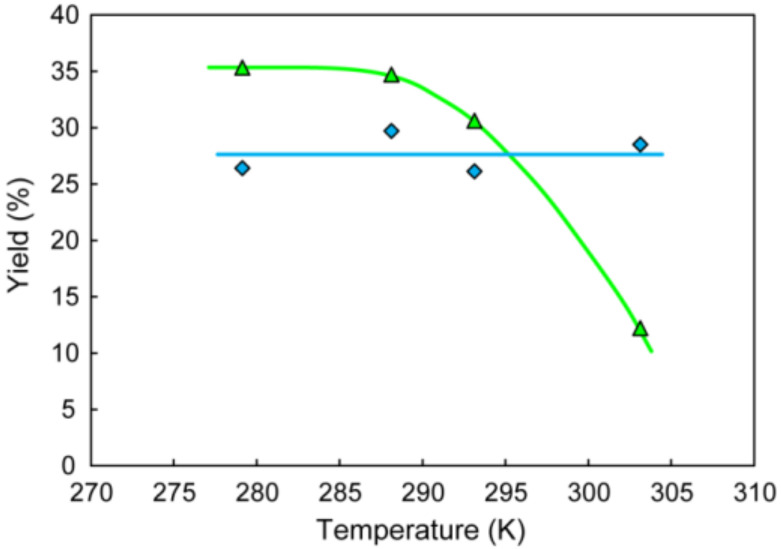
Yield of C_12_py-Rb-Mo_8_ and C_12_py-Cs-Mo_8_ hybrid crystals under various temperatures (blue diamonds: C_12_py-Rb-Mo_8_, green triangles: C_12_py-Cs-Mo_8_).

The yield of C_12_py-Rb-Mo_8_ and C_12_py-Cs-Mo_8_ was investigated under various crystallization conditions. The keeping temperature was changed from 279 to 303 K (Figure 5). The obtained colorless solids or crystals were confirmed to be the C_12_py-Rb-Mo_8_ and C_12_py-Cs-Mo_8_ hybrid crystals by their IR spectra (Figure S4). The yield of C_12_py-Rb-Mo_8_ was almost constant at 25–30% in the considered temperature range. On the other hand, the yield of C_12_py-Cs-Mo_8_ was 35% at 279 K; however, this decreased drastically over 288 K to 12% at 303 K.

## 4. Discussion

The hybrid crystals of C_12_py-Mo_8_ and C_12_py-Mo_8_-AN were pseudopolymorphs (Figure 3). The emergence of the pseudopolymorphism was caused by the difference in the heating temperatures as shown in Scheme 1b. The lower heating temperature (308 K) and smaller difference (5 K) to the keeping temperature (303 K) provided milder crystallization conditions, resulting in the formation of C_12_py-Mo_8_ without crystallization solvent. On the other hand, the higher heating temperature (353 K) and larger difference (50 K) to the keeping temperature (303 K) caused severer crystallization conditions, resulting in the phase of C_12_py-Mo_8_-AN including crystallization solvent. These results suggest that C_12_py-Mo_8_ obtained under milder crystallization conditions was an energetically preferred phase, and that C_12_py-Mo_8_-AN formed under severer conditions was a kinetically preferred phase. This implication is consistent with the results that C_12_py-Mo_8_ was also formed by the gradual reoxidation of C_12_py-red-Mo, which will slowly crystallize C_12_py-Mo_8_. From a structural aspect, the crystal structure and molecular conformation observed in C_12_py-Mo_8_-AN were similar to those in C_16_py-α-Mo_8_ hybrid crystals [39] except for the layer distance and the presence of crystallization solvent, while no similar phase to C_12_py-Mo_8_ was obtained for the C_16_py-Mo_8_ system.

The isomerization of α-Mo_8_ anion to β-Mo_8_ induced the introduction of metal into the hybrid crystals [41]. Rb^+^ and Cs^+^ were successfully incorporated when C_12_py-Mo_8_ was employed as the starting precipitate. The crystal structures and molecular conformations observed in C_12_py-Rb-Mo_8_ and C_12_py-Cs-Mo_8_ were similar to those in C_16_py-Cs-Mo_8_ [41]. The C_16_py-Cs-Mo_8_ hybrid crystal was obtained only as a mixture with low yield (<10%). However, C_12_py-Rb-Mo_8_ and C_12_py-Cs-Mo_8_ were able to isolate as a pure phase with moderate yield (~30%). This will be because of higher solubility of C_12_py-Mo_8_ than C_16_py-Mo_8_ derived from the shorter hydrophobic alkyl chain.

As mentioned above, the C_12_py-Mo_8_ hybrid crystal can incorporate Rb^+^ and Cs^+^ cations from the solution phase into the solids to form the C_12_py-Rb-Mo_8_ and C_12_py-Cs-Mo_8_ hybrid crystals. This property renders it possible for utilizing C_12_py-Mo_8_ as absorbers capturing radioactive metal cations. Figure 5 shows the yields of C_12_py-Rb-Mo_8_ and C_12_py-Cs-Mo_8_ were approximately 30% based on the solid RbNO_3_ or CsNO_3_ in maximum, which suggests that approximately 30% of Rb^+^ or Cs^+^ could be removed from the solution phase. The removal of Rb^+^ and Cs^+^ will be an irreversible process: Rb^+^ and Cs^+^ in the solution phase were incorporated into the solid state, resulting in C_12_py-Rb-Mo_8_ and C_12_py-Cs-Mo_8_ crystals with rigid packing and low solubility. Therefore, the captured Rb^+^ and Cs^+^ could not be facilely released, and it seems difficult to reuse C_12_py-Mo_8_ as a metal-cation capturing material.

The moderate capturing rate (ca. 30%) of C_12_py-Mo_8_ will be due to the formation mechanism of C_12_py-Rb-Mo_8_ and C_12_py-Cs-Mo_8_ (Scheme 1a). The α-Mo_8_ anions dissolved from the starting C_12_py-Mo_8_ solid isomerized to β-Mo_8_ in acetonitrile, and sequentially reprecipitated with metal cations to form C_12_py-Rb-Mo_8_ and C_12_py-Cs-Mo_8_. The entire process is not a solid-state ion-exchange reaction, and less superior to well-established solid-state systems regarding the exchange rate and selectivity [11,12,13]. Changing the C_12_py cation to an ionic-liquid surfactant may improve the capturing rate. Although the capturing property of C_12_py-Mo_8_ is not sufficient as toxic metal cation-capturing materials, the results presented here may open a new possibility of POM–surfactant hybrid materials.

## Figures and Tables

**Table 1:** Crystallographic data.

Compound	C_12_py-Mo_8_	C_12_py-Mo_8_-AN	C_12_py-Rb-Mo_8_	C_12_py-Cs-Mo_8_
Chemical formula	C_68_H_120_N_4_Mo_8_O_26_	C_70_H_123_N_5_Mo_8_O_26_	C_34_H_60_N_2_Rb_2_Mo_8_O_26_	C_34_H_60_N_2_Cs_2_Mo_8_O_26_
Formula weight	2177.23	2218.28	1851.30	1946.18
Crystal system	triclinic	triclinic	triclinic	triclinic
Space group	*P*$\bar{1}$ (No. 2)	*P*$\bar{1}$ (No. 2)	*P*$\bar{1}$ (No. 2)	*P*$\bar{1}$ (No. 2)
*a* (Å)	10.3430(5)	12.8469(8)	7.8864(4)	7.9663(3)
*b* (Å)	11.3843(6)	18.0395(10)	10.6302(4)	17.4100(5)
*c* (Å)	19.7519(11)	20.5427(12)	17.7431(10)	20.3300(6)
*α* (°)	79.308(5)	108.6372(12)	91.918(3)	75.492(3)
*β* (°)	76.042(4)	94.6749(15)	98.1055(19)	88.785(3)
*γ* (°)	70.315(4)	90.4647(14)	111.357(3)	85.313(3)
*V* (Å^3^)	2111.3(2)	4493.2(5)	1365.68(12)	2720.59(16)
*Z*	1	2	1	2
*ρ*_calcd_ (g∙cm^−3^)	1.712	1.639	2.251	2.376
*T* (K)	93	193	193	193
Wavelength (Å)	0.71073	0.71075	0.71075	0.71073
*μ* (mm^−1^)	1.219	1.148	3.619	3.178
No. of reflections measured	32,803	66,871	22,036	21,492
No. of independent reflections	16,306	20,485	6242	12,476
*R*_int_	0.0830	0.0485	0.0485	0.0445
No. of parameters	480	1315	418	652
*R*_1_ (*I* > 2*σ*(*I*))	0.0554	0.0494	0.0313	0.0543
*wR*_2_ (all data)	0.0906	0.0904	0.0606	0.1395

## Data Availability

Further details of the crystal structure investigation (CCDC 2153845-2153849) can be obtained free of charge via www.ccdc.cam.ac.uk/data_request/cif (accessed on 22 February 2022), or by emailing data_request@ccdc.cam.ac.uk, or by contacting The Cambridge Crystallographic Data Centre, 12 Union Road, Cambridge CB2 1EZ, UK; Fax: +44-1223-336033.

## Supplementary Materials

The following supporting information can be downloaded at: https://www.mdpi.com/article/10.3390/ma15072429/s1, Table S1: Crystallographic data for C_12_py-Mo_8_ prepared from C_12_py-red-Mo; Figure S1: Measured and calculated powder X-ray diffraction patterns of C_12_py-Mo_8_ and related hybrid crystals. Measured patterns were obtained at ambient temperature. Calculated patterns were obtained from the structure revealed by single-crystal X-ray diffraction; Figure S2: Structural information of C_12_py-Mo_8_ hybrid crystal prepared by the reoxidation of C_12_py-red-Mo; Figure S3: Crystal structure of C_12_py-Mo_8_ hybrid crystal prepared by the reoxidation of C_12_py-red-Mo; Figure S4: IR spectra of metal cation-introduced C_12_py-Mo_8_ hybrid crystals obtained at various crystallization temperatures.
