# Supplementary material for: Polyoxomolybdate Layered Crystals Constructed from a Heterocyclic Surfactant: Syntheses, Pseudopolymorphism and Introduction of Metal Cations
Source: Materials (Basel). 2022 Mar 25;15(7):2429. doi: 10.3390/ma15072429 (PMC8999574; doi:10.3390/ma15072429)
Supplement: Supplementary file 1 [file materials-15-02429-s001.zip › materials-1637255-supplementary.pdf]

*Supporting Information*

# Polyoxomolybdate Layered Crystals Hybridized with a Heterocyclic Surfactant: Syntheses, Pseudopolymorphism and Introduction of Metal Cations

## 1. Synthesis and Characterization of $C_{12}$py-$Mo_8$ by the Reoxidation of Reduced POM

### *1.1. Synthesis*

$C_{12}$py-$Mo_8$ was synthesized by another method utilizing gradual reoxidation of reduced molybdenum POM hybridized with $C_{12}$py cation ($C_{12}$py-red-Mo). The $C_{12}$py-red-Mo hybrid was prepared as follows; $Na_2MoO_4 \cdot 2H_2O$ (2.6 g, 11 mmol) was dissolved in $H_2O$ (10 mL), and the pH level was adjusted to 4.0 with 6 M HCl. To this colorless solution was added solid ascorbic acid (0.1 g, 0.5 mmol) as reducing reagent. After stirring for 40 min, the solution color turned dark blue indicating the formation of reduced POM species, and sequentially a water/ethanol (10 mL, 1:2 (v/v)) solution of $C_{12}$pyCl·$H_2O$ (0.42 g, 1.5 mmol) was added to the solution with stirring for 10 min. Resultant suspension was filtered to obtain blue precipitate of $C_{12}$py-red-Mo. Colorless block crystals of $C_{12}$py-$Mo_8$ was grown by slow evaporation of *N*,*N*-dimethylformamide/acetone (15 mL, 2:1 (v/v)) solution of $C_{12}$py-red-Mo. CHN elemental analysis: Calcd for $C_{74}H_{138}N_4Mo_8O_{29}$: C: 38.39, H: 6.01, N: 2.42%. Found: C: 38.93, H: 5.74, N: 2.58%. IR (KBr disk): 954 (w), 927 (s), 914 (s), 844 (m), 823 (m), 800 (m), 779 (m), 713 (m), 685 (m), 663 (m), 555 (w), 525 (w), 480 (w), 456 (w), 411 (w) $cm^{-1}$.

### *1.2. Crystal Structure Deteramination*

Diffraction data were collected and processed on a Rigaku R-AXIS RAPID diffractometer by using graphite monochromated Mo K$\alpha$ radiation with PROCESS-AUTO. The diffraction data recorded at the 2D beamline in the Pohang Accelerator Laboratory (PAL, South Korea) confirmed the same crystal structure. Crystal structure was solved by SUPERFLIP, and refined by the full-matrix least-squares using SHELXL (Version 2018/3) through CrystalStructure software package. Non-hydrogen atoms were refined anisotropically, and the hydrogen atoms on C atoms were located in calculated positions. CCDC 2153849.

## 2. Structure of $C_{12}$py-$Mo_8$ by the Reoxidation of Reduced POM

Gradual reoxidation of initially blue solution containing $C_{12}$py-red-Mo endowed colorless single crystals of $C_{12}$py-$Mo_8$. The IR spectrum and powder XRD pattern of the obtained crystal (Figure S2) were similar to those of the $C_{12}$py-$Mo_8$ precipitate and crystal prepared from conventional method using $Na_2MoO_4 \cdot 2H_2O$ or $(NH_4)_6Mo_7O_{24} \cdot 4H_2O$ as a molybdenum source (Figures 1a, 1b, 2a, 2b). In Figure S2b, the powder XRD patterns measured for obtained crystal and calculated from the result of crystal structure analysis were slightly different in the peak position and intensity probably because of pre-ferred orientation derived from the distinct layered structures of $C_{12}$py-$Mo_8$. In addition, the crystal structure of $C_{12}$py-$Mo_8$ obtained from $C_{12}$py-red-Mo (Table S1, Figure S3) was the same as that of the $C_{12}$py-$Mo_8$ crystal prepared from the conventional method (Figure 3a, 3c).

**Table S1.** Crystallographic data for $C_{12}$py-$Mo_8$ prepared from $C_{12}$py-red-Mo.

| Compound | $C_{12}$py-$Mo_8$ |
|---|---|
| Chemical formula | $C_{68}H_{120}N_4Mo_8O_{26}$ |
| Formula weight | 2177.23 |
| Crystal system | triclinic |
| Space group | $P\bar{1}$ (No. 2) |
| $a$ (Å) | 10.4334(6) |
| $b$ (Å) | 11.6760(6) |
| $c$ (Å) | 20.1877(10) |
| $\alpha$ (°) | 77.7581(14) |
| $\beta$ (°) | 75.7242(15) |
| $\gamma$ (°) | 68.5309(14) |
| $V$ (Å$^3$) | 2197.64(19) |
| $Z$ | 1 |
| $\rho_{calcd}$ (g cm$^{-3}$) | 1.645 |
| $T$ (K) | 296 |
| Wavelength (Å) | 0.71075 |
| $\mu$ (mm$^{-1}$) | 1.171 |
| No. of reflections measured | 35543 |
| No. of independent reflections | 10042 |
| $R_{int}$ | 0.0642 |
| No. of parameters | 525 |
| $R_1$ ($I > 2\sigma(I)$) | 0.0546 |
| $wR_2$ (all data) | 0.1416 |

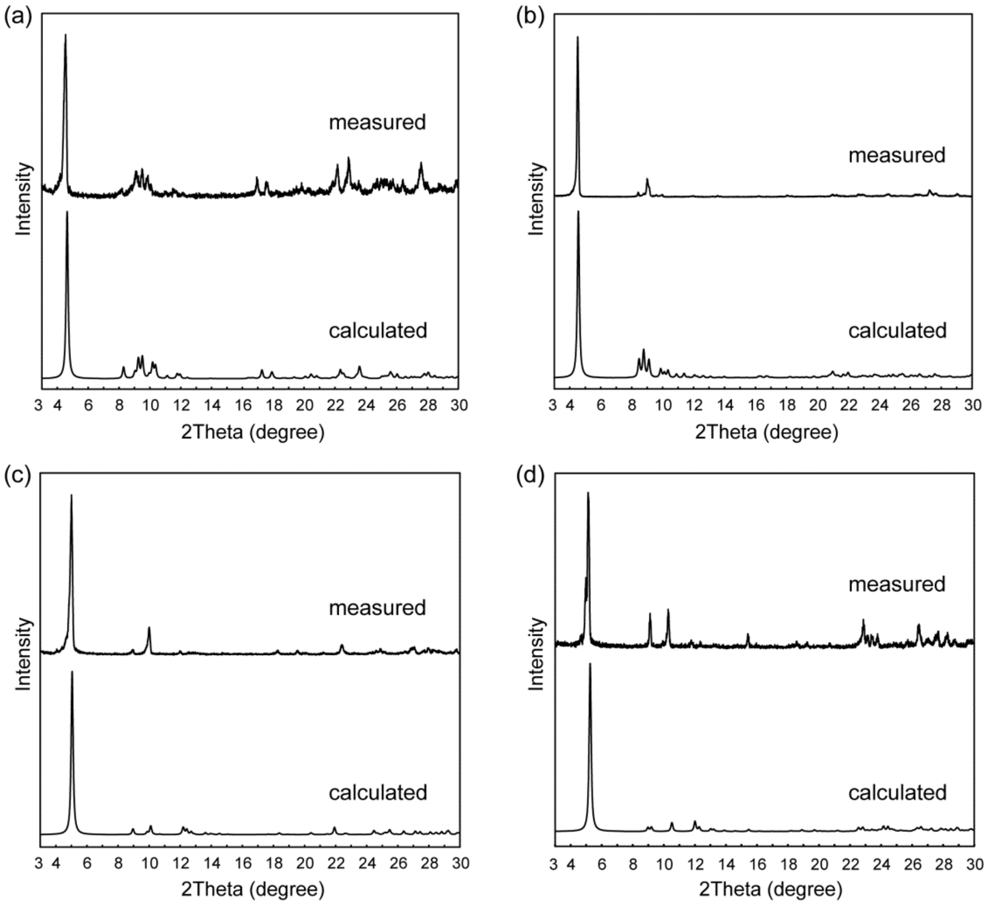


**Figure S1.** Measured and calculated powder X-ray diffraction patterns of $C_{12}$py-$Mo_8$ and related hybrid crystals. Measured patterns were obtained at ambient temperature. Calculated patterns were obtained from the structure revealed by single-crystal X-ray diffraction: (**a**) $C_{12}$py-$Mo_8$; (**b**) $C_{12}$py-$Mo_8$-AN; (**c**) $C_{12}$py-Rb-$Mo_8$; (**d**) $C_{12}$py-Cs-$Mo_8$.

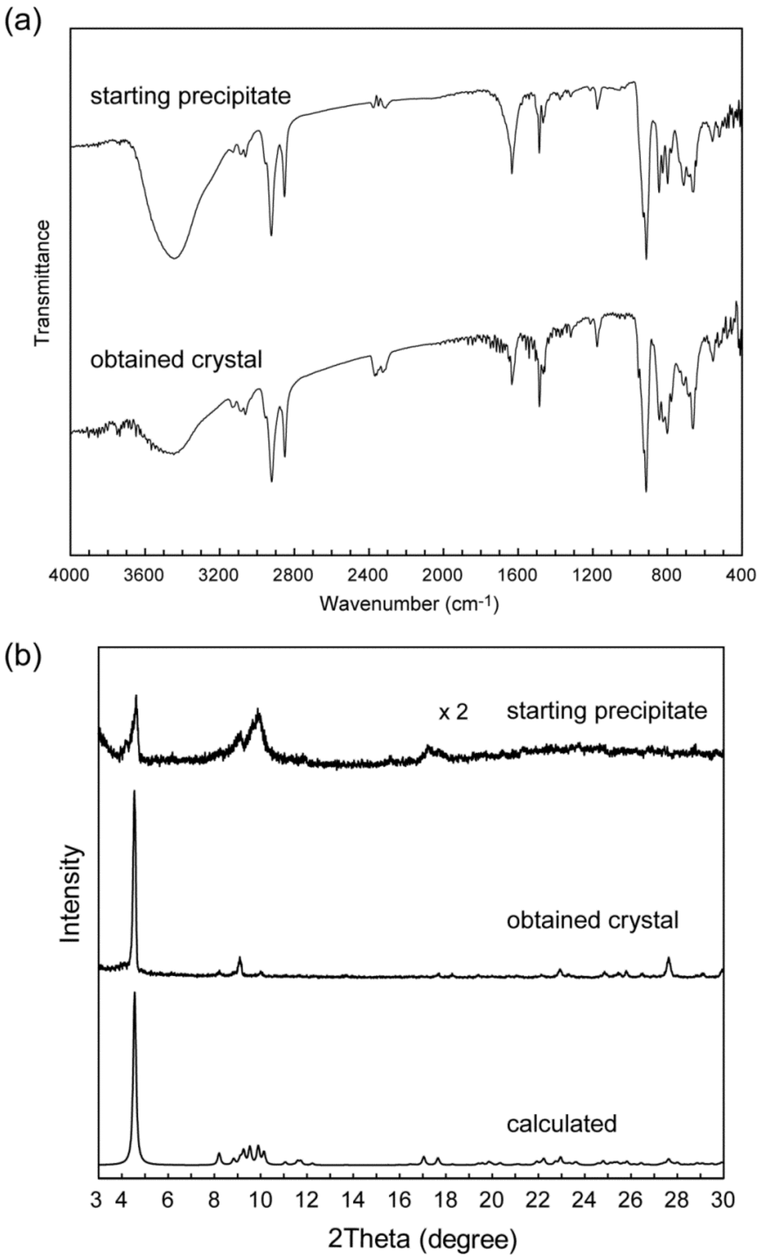


**Figure S2.** Structural information of $C_{12}$py-$Mo_8$ hybrid crystal prepared by the reoxidation of $C_{12}$py-red-Mo: (**a**) IR spectra of starting precipitate and obtained crystal; (**b**) Measured powder XRD patterns of starting precipitate and obtained crystal together with the pattern calculated from the structure revealed by single-crystal X-ray diffraction.

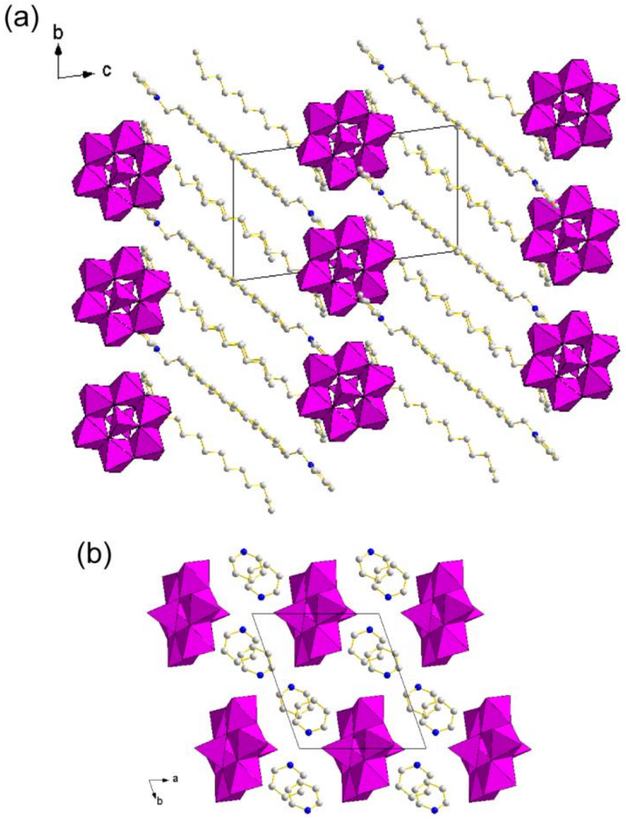


**Figure S3.** Crystal structure of $C_{12}$py-$Mo_8$ hybrid crystal prepared by the reoxidation of $C_{12}$py-red-Mo (C: gray, N: blue; $\alpha$-$Mo_8$ in purple polyhedrons). H atoms and disordered atoms are omitted for clarity: (**a**) Packing diagram of $C_{12}$py-$Mo_8$ along the *a* axis; (**b**) Molecular arrangements in the inorganic layers (*ab* plane).

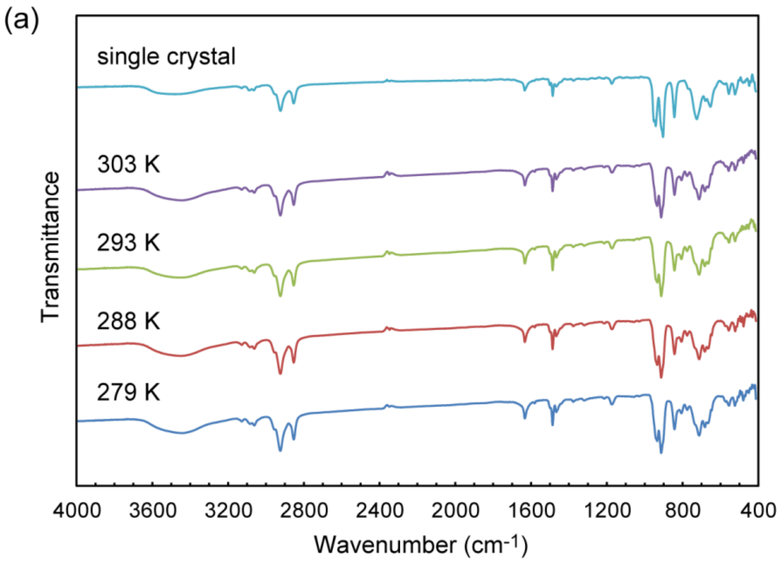


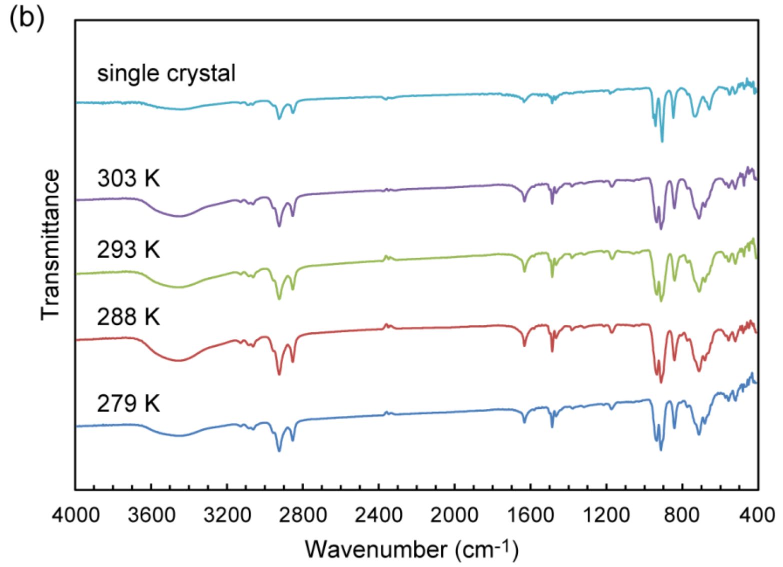


**Figure S4.** IR spectra of metal cation-introduced $C_{12}$py-$Mo_8$ hybrid crystals obtained at various crystallization temperatures: (**a**) $C_{12}$py-Rb-$Mo_8$; (**b**) $C_{12}$py-Cs-$Mo_8$.
